# Enhancing Nurse Leaders Evidence‐Based Practice Implementation Leadership Competence. A Systematic Review and Meta‐Analysis of Educational Interventions

**DOI:** 10.1111/wvn.70164

**Published:** 2026-07-13

**Authors:** Jaakko Varpula, Riitta Askola, Desale Tewelde Kahsay, Anna Axelin, Laura Virtanen, Riikka Maijala, Laura‐Maria Peltonen

**Affiliations:** ^1^ Department of Nursing Science University of Turku Turku Finland; ^2^ Department of Anaesthesia and Intensive Care University of Turku Turku Finland; ^3^ The Wellbeing Services County of Southwest Finland Turku Finland; ^4^ Department of Health and Social Management University of Eastern Finland Kuopio Finland; ^5^ The Wellbeing Services Country of North Savo Kuopio Finland

**Keywords:** continuing, education, evidence‐based practice, leadership, nursing

## Abstract

**Background:**

Nurse leaders play a pivotal role in facilitating evidence‐based practice (EBP). Gaps persist in their competence and engagement in EBP implementation leadership. Educational interventions targeted at nurse leaders can improve their EBP competencies.

**Aim:**

To evaluate the effectiveness of educational interventions on nurse leaders' competence in EBP implementation leadership and organizational, staff, and patient‐related outcomes.

**Methods:**

A comprehensive search was conducted across major databases and trial registries in September 2024. Six intervention studies met the inclusion criteria, encompassing various healthcare settings and leadership roles. Data were synthesized narratively and analyzed statistically using meta‐analysis.

**Results:**

Educational interventions significantly improved nurse leaders' implementation leadership (SMD = 0.74), EBP competence (SMD = 1.76), and knowledge (SMD = 1.05). Organizational readiness (SMD = 0.65) and implementation climate (SMD = 1.60) also showed positive effects. The overall certainty of evidence was rated low due to methodological limitations.

**Linking Evidence to Action:**

Educational interventions targeting nurse leaders are effective in enhancing EBP implementation leadership competencies and fostering supportive organizational environments. Investing in educational programs for nurse leaders may strengthen EBP implementation leadership, implementation climate, and organizational readiness.

## 1 Introduction

Evidence‐based practice (EBP) is the foundation of modern healthcare (Mackey and Bassendowski 2017). EBP is the use of best available evidence, clinical expertise, and consideration of patient preferences to make clinical decisions on care (Melnyk and Fineout‐Overholt 2023). A gap has been identified between implementing research evidence into nursing practice (Leach and Tucker 2018). Organizations use different strategies to enhance the implementation of EBP (Mathieson et al. 2018), of which multicomponent strategies are most effective (Cassidy et al. 2021). Structured strategies and models provide essential guidance for EBP implementation; their effectiveness relies on organizational dynamics and leadership engagement—particularly nurse leaders' operationalization of these approaches within clinical settings (Chays‐Amania et al. 2025).

The central role of the nurse leader, as facilitator for EBP implementation has been well established (Speroni et al. 2020). Nurse leaders facilitate implementation of EBP through encouragement, leading by example (Mathew et al. 2024), mentoring (Agnel et al. 2025), implementation leadership (specific and strategic behaviors by leaders to implement EBP) (Aarons et al. 2014), and fostering a supportive implementation climate (shared perceptions among employees how EBP is expected, supported and rewarded) in the unit (Weiner et al. 2011). Yet, disparities lie in their actualization of that central role. A survey of nurses showed nurse leaders do not communicate the importance of EBP or facilitate its practice (Lunden et al. 2021). One challenge in facilitating EBP is either leaders' or organizations' low priority towards EBP (Melnyk et al. 2016; Sebire et al. 2025), organizational barriers (e.g., time constraints, competing priorities, limited resources, policies, lack of support) as well as inadequate competence in EBP (Koivunen et al. 2024; Sebire et al. 2025) and EBP implementation leadership (Hu et al. 2025).

Educational interventions are essential for nurse leaders (Page, Halcomb, and Sim 2021; Page, McKenzie, et al. 2021). Nevertheless, most are targeted to staff. There is an abundance of systematic reviews on the effectiveness of EBP educational interventions in nurses (Portela Dos Santos et al. 2022), but no systematic reviews assess the effectiveness of educational interventions targeted at nurse leaders. This systematic review aims to fill this knowledge gap by synthesizing the existing available evidence on the effectiveness of EBP educational interventions aimed at improving nurse leaders' EBP implementation leadership competence, and their effect on patients', staff's,' and organizations' outcomes. The review questions are as follows:

1. What is the effectiveness of educational interventions aiming to improve nurse leaders' EBP implementation leadership competence?
2. What is the effectiveness of educational interventions aiming to improve nurse leaders' EBP implementation leadership on patient, staff, and organizational outcomes?

## 2 Materials and Methods

A systematic review and meta‐analysis were conducted following the Preferred Reporting Items for Systematic Reviews and Meta‐Analyses (PRISMA) guidelines (Page, Halcomb, and Sim 2021; Page, McKenzie, et al. 2021). Meta‐analysis was undertaken to quantitatively synthesize evidence across studies, enhance statistical power, and provide more precise and generalizable effect estimates than individual studies alone. The study protocol was registered in the PROSPERO database (CRD42024579239).

### 2.1 Eligibility Criteria

The eligibility criteria for this systematic review were formulated using the PIOS (population, intervention, outcome, study types) framework (Table 1) (Miller and Forrest 2001).

**TABLE 1:** PIOS.

Population	Intervention	Outcome	Study type(s)
Nurse leaders	Educational interventions	Competence in EBP implementation leadership	Intervention studies

We included peer‐reviewed original studies in experimental designs (randomized controlled trials, crossover studies, quasi‐experimental). Pilot, feasibility, adaptive, and pragmatic trials were excluded. We included studies with nurse leaders, defined as professionals responsible for nursing practice who have an official managerial or administrative role in the organization. Educational interventions such as formal education, continuous education, mentoring, onboarding, and instructions which aim to improve nurse leaders' competence in leading EBP implementation were included.

Our primary outcome was the effects on nurse leaders' competence in EBP implementation leadership, defined as the process of leading the integration of the best available research evidence, clinical expertise, and patient values to inform patient care as well as creating a supportive culture, education, and overcoming systemic barriers (Ominyi et al. 2025). We used the following definition of competence: *the habitual and judicious use of communication, knowledge, technical skills, clinical reasoning, emotions, values, and reflection in daily practice for the benefit of the individual and community being served* (Epstein and Hundert 2002).

Our secondary outcomes were other effects of the interventions reported in the studies for nurse leaders (e.g., behavior, self‐efficacy), healthcare staff (e.g., attitudes towards EBP, job satisfaction), and organization (e.g., organizational readiness, work environment).

### 2.2 Search Strategy, Data Sources, and Screening

The literature search was conducted in September 2024 to PubMed, CINAHL, Scopus, Web of Science, and the Cochrane Library. We also searched the clinical trial databases (ClinicalTrials.gov, WHO International Clinical Trials Registry Platform, EU Clinical Trials Register). The database search was supplemented with a manual search.

Database searches were conducted with the following search string (*Nurse leader* AND *Evidence‐based practice* AND *Educational intervention* AND *Competence*). A librarian confirmed the appropriateness of the search. The complete search strings for each database are described in Supporting Information S1.

**FIGURE 1:**
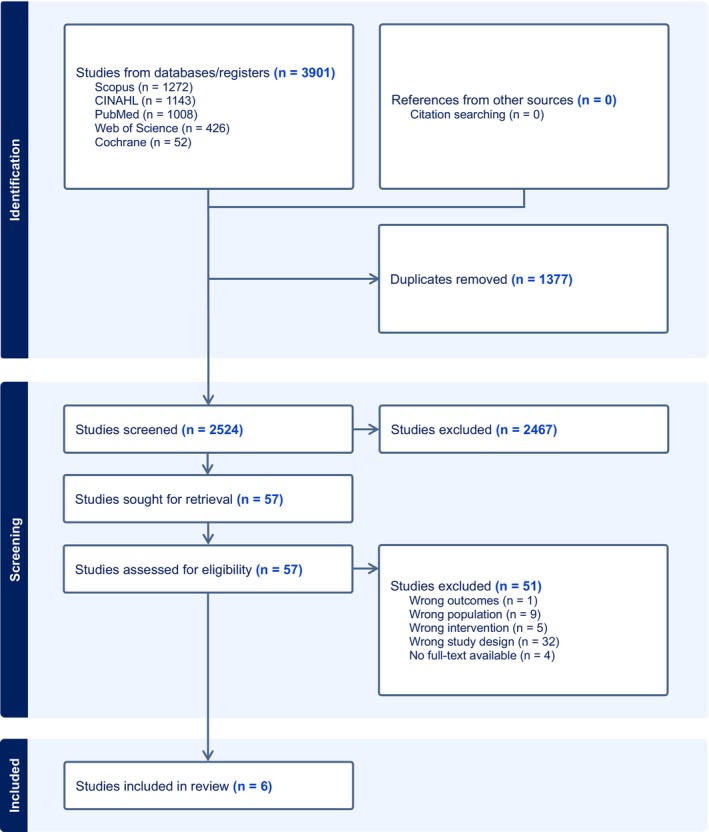
PRISMA 2020 flow diagram (Page, Halcomb, and Sim 2021; Page, McKenzie, et al. 2021).

References were exported from the databases to Covidence where duplicates were removed. Two reviewers (J.V. and R.A.) performed the screening process of studies independently. A third reviewer (R.M. or L.V.) solved disagreements in the screening. The screening and selection process is described in Figure 1.

### 2.3 Data Extraction Process

Data extracted from the studies were information on the publication (authors, publication year, country), purpose of the study, setting, sample, methodology, outcome variables, description of the intervention, and results. We included all results, whether they were statistically significant or not.

### 2.4 Risk of Bias Assessment

The risk of bias of the studies was assessed using Cochrane collaboration risk of bias tools. For randomized controlled trials, RoB 2.0 was used (Sterne et al. 2019). Risk of bias assessment for non‐randomized studies of interventions was conducted using the ROBINS‐I tool (Sterne et al. 2016). Risk of bias assessment was conducted by J.V., R.M., and L.V.

### 2.5 Data Synthesis

Narrative synthesis and meta‐analysis were used to summarize the findings of the individual studies. A narrative synthesis was conducted to describe how, why, and for whom the intervention works.

A meta‐analysis was conducted using standardized mean differences (SMD) with 95% confidence intervals to estimate the effects of the educational interventions. SMD is used to summarize data statistically in situations where studies included in a systematic review assess the same outcome with different scales or instruments (Higgins et al. 2024). RevMan meta‐analysis software, which uses Hedge's *g*, was used to analyze the data. Hedge's *g* method is a suitable option when dealing with small sample sizes and outcomes measured with different scales across studies (Higgins et al. 2024). The effect sizes were interpreted according to Cohen's *d* values guidelines; *d* values of 0.2 are considered a small effect, 0.5 a medium effect, and 0.8 a large effect (Cohen 1988). Studies used multiple time points to measure the effect; baseline and final time points were used to analyze the effect of the intervention. In randomized trial studies, only the intervention group was included in the meta‐analysis.

### 2.6 Confidence in the Body of Evidence

We used the Grading of Recommendations, Assessment, Development, and Evaluations (GRADE) framework to assess the quality of the evidence. The assessment considers the risk of bias, imprecision, inconsistency, indirectness, and publication bias (Guyatt et al. 2008).

## 3 Results

### 3.1 Characteristics of Included Studies

The studies employed a variety of research designs. Most were pre‐post designs (Gifford et al. 2014; Lavoie‐Tremblay et al. 2012; Patton et al. 2024; Proctor et al. 2019). Only one study was a randomized controlled trial (Tucker et al. 2022). A diverse range of nurse leaders across various social and healthcare settings, including tertiary hospital care (Patton et al. 2024) and community healthcare (Gifford et al. 2014), were targeted. Sample sizes ranged from 14 (Caramanica and Spiva 2018) to 88 (Tucker et al. 2022).

The educational interventions employed various approaches and delivery methods. Most incorporated a combination of didactic training and practical exercises (Caramanica and Spiva 2018; Gifford et al. 2014; Lavoie‐Tremblay et al. 2012; Patton et al. 2024; Proctor et al. 2019; Tucker et al. 2022), Organizational and external support were provided in some (Caramanica and Spiva 2018; Gifford et al. 2014; Lavoie‐Tremblay et al. 2012; Proctor et al. 2019). The key components of the interventions are described in Table 2.

**TABLE 2:** Characteristics of included studies.

Author (year), country	Setting	Design	Sample	Intervention	Intervention key components	Control
Caramanica and Spiva (2018) United States	Acute care	Mixed methods	Nurse managers (*n* = 14), Clinical nurses (*n* = 15), Evidence‐based practice mentors (*n* = 10).	Educational intervention (EBP Education and EBP Toolkit)	Two focused workshops; leadership behavior training; EBP toolkit for data support; role clarity for nurse managers	—
Gifford et al. (2014) Canada	Community‐based healthcare	Pre‐post study	Management and clinical leaders (*n* = 32)	Multifaceted EIDM intervention	Single workshop; ongoing support from evidence facilitators; access to resources; peer sharing and recognition activities	—
Lavoie‐Tremblay et al. (2012) Canada	Multiple organizations	Pre‐post study	Executive nurses (*n* = 34)	Educational intervention (EXTRA program)	Modular residency sessions; organizational intervention projects; e‐learning; mentoring; post‐program support and networking	—
Patton et al. (2024) United States	Tertiary, pediatric	Pre‐post study	Nursing leaders (*n* = 34), clinical nurses (*n* = 51)	EBP Leadership Behavior Program (LBP)	Three‐part workshop series; blended learning; guided leadership planning; based on Iowa Model and EBP competencies	—
Proctor et al. (2019) United States	Substance‐abuse	Pre‐post study	Training participants (Clinical managers, quality improvement coordinators, program directors, *n* = 16)	Training in Implementation Practice Leadership (TRIPLE)	Structured training with lectures, exercises, and readings; hands‐on clinic projects; peer networking; expert‐led coaching	—
Tucker et al. (2022) United States	Cancer center	Randomized waitlist‐controlled trial	Nurse leaders (*n* = 88)	EBP leadership immersion intervention	Intensive 5‐day immersion using the ARCC model; experiential learning; standardized curriculum; real‐world issue application; trained facilitators	Wait‐list

The dependent variables for the meta‐analysis were measured with a variety of self‐reported scales. Knowledge was measured in the studies with EBP Knowledge Scale (Tucker et al. 2022), Knowledge gained from the EXTRA program, and Knowledge applied from the EXTRA program (Lavoie‐Tremblay et al. 2012). EBP competence was measure with EBP Nurse Leadership Scale (Caramanica and Spiva 2018), Nursing Manager EBP Competency Scale (Patton et al. 2024), and EBP Competency Scale (Tucker et al. 2022). Implementation leadership was measure with Implementation Leadership Scale (ILS) (Patton et al. 2024; Proctor et al. 2019), EBP Implementation Scale (Tucker et al. 2022), and EBP Implementation Strategies Self‐Efficacy Scale (ISE4EB) (Tucker et al. 2022). Implementation climate was measured with EBP Work Environment Scale (Caramanica and Spiva 2018) and Implementation Climate Scale (ICS) (Proctor et al. 2019) whereas organizational readiness was measured with Organizational Readiness for Implementing Change (ORIC) (Proctor et al. 2019) and Organizational Culture and Readiness System‐wide Integration of Evidence‐based Practice Scale (OCRSIEP) (Tucker et al. 2022).

#### 3.1.1 A Theory of How Why and for Whom the Intervention Works

Interventions shared a common goal of enhancing leadership competencies and fostering a supportive environment for EBP implementation. However, the mechanism behind the intervention effects differed. Caramanica and Spiva (2018) focused on cultivating a shared vision for EBP based on theories of supportive leadership and team dynamics. The EIDM intervention employed organizational strategies based on theories of organizational behavior to promote evidence‐informed decision making among nurse leaders (Gifford et al. 2014). The Executive Training for Research Application (EXTRA) incorporated residency sessions and mentoring, emphasizing the role of experiential learning and social networks in enhancing EBP knowledge and use (Lavoie‐Tremblay et al. 2012). In the Evidence‐Based Practice Leadership Behavior Program (EBP LBP), theories of adult learning and leadership behavior were utilized (Patton et al. 2024). The Training in Implementation Practice Leadership (TRIPLE) intervention leveraged theories of proactive behavior and organizational readiness (Proctor et al. 2019). Whilst Tucker et al. (2022) utilized hands‐on learning grounded in theories of self‐efficacy and implementation science.

### 3.2 Risk of Bias

**FIGURE 2:**
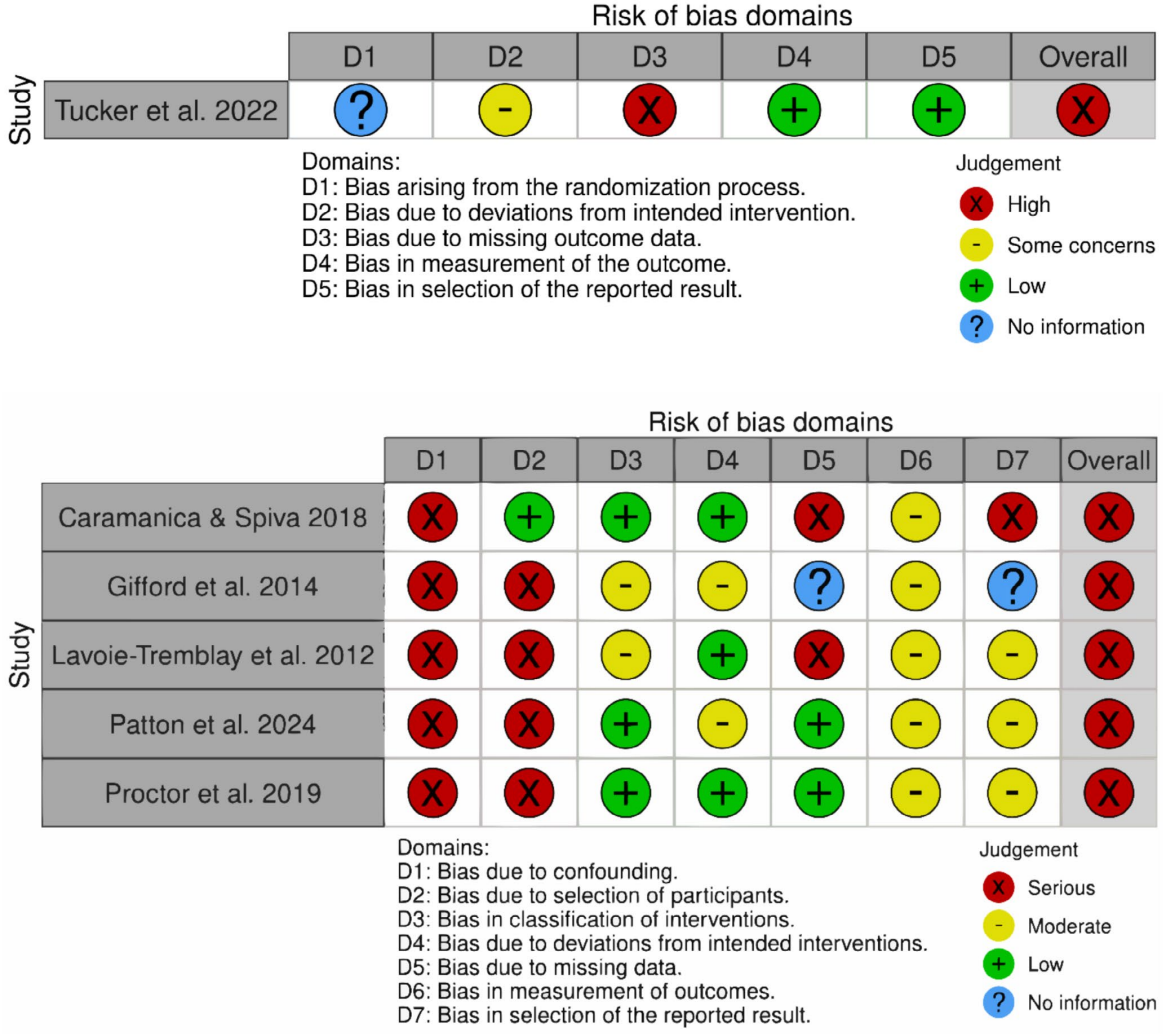
Risk of bias assessments.

The overall risk of bias was serious in Tucker et al. (2022) and all the non‐randomized studies (Caramanica and Spiva 2018; Gifford et al. 2014; Lavoie‐Tremblay et al. 2012; Patton et al. 2024; Proctor et al. 2019), mostly due to confounding and selection of participants (Figure 2).

### 3.3 Effectiveness of Educational Interventions

**FIGURE 3:**
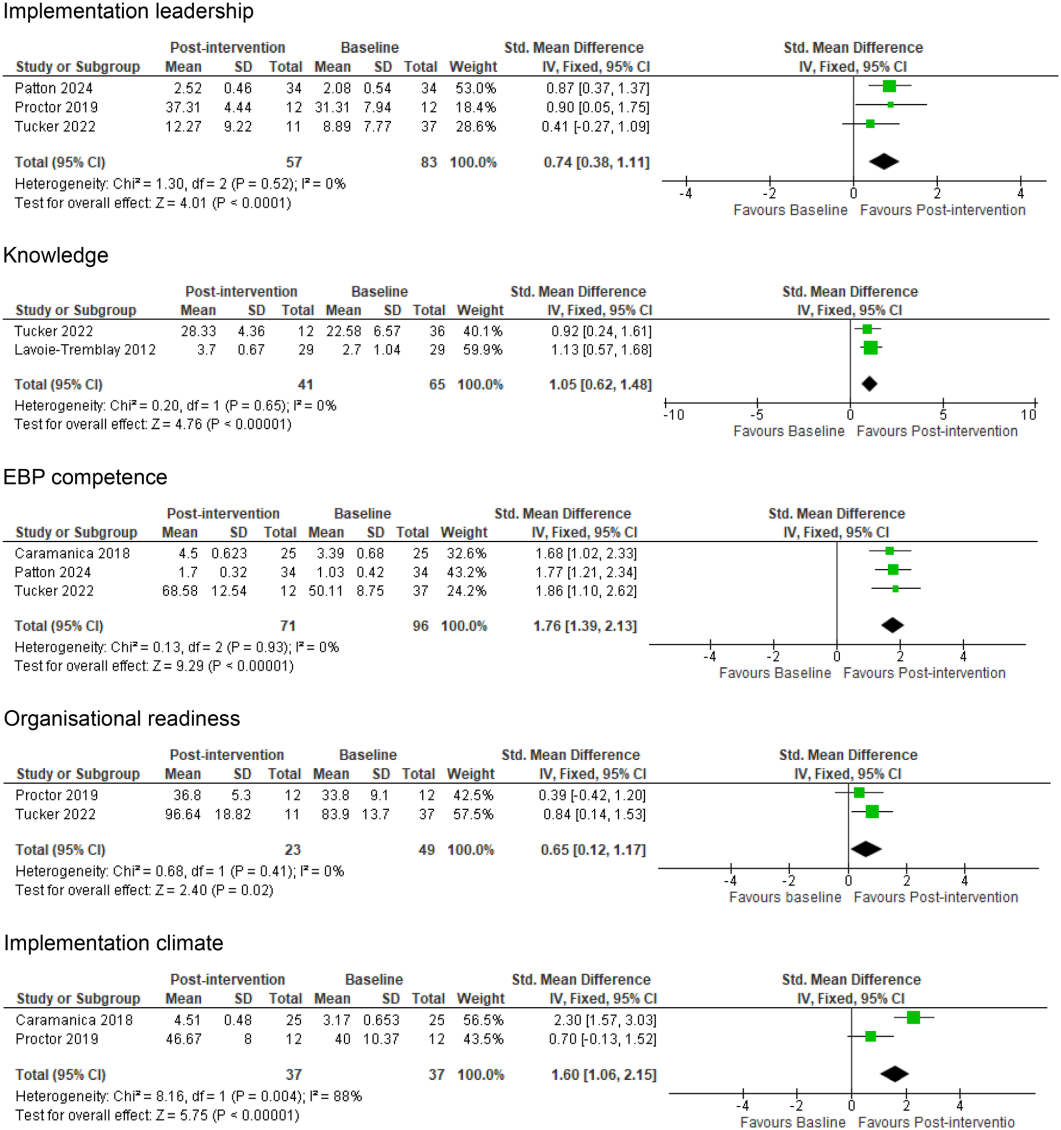
Forest plot for the primary and secondary outcomes.

Data for the effectiveness of educational interventions were summarized using meta‐analysis (Figure 3) between baseline and post‐intervention (pretest, post‐test: Patton et al. 2024; first session, last session: Proctor et al. 2019; baseline, week 104: Tucker et al. 2022; pre‐evaluation, post‐evaluation: Lavoie‐Tremblay et al. 2012; preintervention, post‐intervention: Caramanica and Spiva 2018). Three studies assessed the effect of the educational intervention on implementation leadership. A meta‐analysis of these three studies revealed a statistically significant improvement favoring the intervention, with a SMD of 0.74 (95% CI: 0.38.1.11; *p* < 0.0001). Similarly, three studies evaluated the impact of the educational intervention on the EBP competence of nursing leadership. The post‐intervention value was significantly higher than the baseline value, with a SMD of 1.76 (95% CI: 1.39, 2.13; *p* < 0.00001). Two studies assessed the impact of the intervention on leaders' knowledge, revealing a significant difference in favor of the intervention, with a SMD of 1.05 (95% CI: 0.62, 1.48; *p* < 0.00001). In all primary outcomes, the educational intervention enhanced competence in EBP implementation leadership, with a moderate effect in implementation leadership and a substantial effect in knowledge and EBP competence.

Secondary outcomes (Figure 3), organizational readiness and implementation climate, were pooled for meta‐analysis. Two studies assessed organizational readiness, and the meta‐analysis indicated a significant improvement, with a SMD of 0.65 (95% CI: 0.12, 1.17, *p* = 0.02), favoring the educational intervention. Similarly, two studies evaluated the effect of the intervention on implementation climate, which demonstrated significant improvement with a SMD of 1.60 (95% CI: 1.06, 2.15; *p* < 0.0001), favoring the intervention.

The meta‐analysis demonstrated that educational interventions significantly improved nurse leaders' implementation leadership (SMD 0.74), EBP competence (SMD 1.76), knowledge (SMD 1.05), organizational readiness (SMD 0.65), and implementation climate (SMD 1.60). However, the certainty of evidence for all outcomes was rated as low (Table 3) due to serious risk of bias across studies—most of which were non‐randomized—as well as concerns regarding inconsistency, imprecision, and potential publication bias.

**TABLE 3:** Summary of findings table for main and secondary outcomes.

Outcome	No. of studies	Effect estimate (SMD, 95% CI)	Certainty of evidence (GRADE)
Implementation leadership	3	0.74 (0.38, 1.11)	⨁⨁◯◯ Low
EBP competence	3	1.76 (1.39, 2.13)	⨁⨁◯◯ Low
Knowledge	2	1.05 (0.62, 1.48)	⨁⨁◯◯ Low
Organizational readiness	2	0.65 (0.12, 1.17)	⨁⨁◯◯ Low
Implementation climate	2	1.60 (1.06, 2.15)	⨁⨁◯◯ Low

#### 3.3.1 Outcomes Not Included in Meta‐Analysis

Several outcomes were reported either in only one study or lacked sufficient data for meta‐analysis. These outcomes were summarized narratively. *Evidence‐based nursing practice* was assessed in one study using the Evidence‐Based Nursing Practice Questionnaire, which found a non‐significant mean difference of 0.33 (−0.11, 0.77 95% CI) between baseline and post‐intervention (Caramanica and Spiva 2018). *Evidence‐based practice beliefs* were reported in one study (Tucker et al. 2022), using the EBP Beliefs Scale. A mean difference of 8.75 (3.33, 14.17 95% CI, *p* < 0.0016) was found between baseline and post‐intervention. I*mplementation self‐efficacy* assessed with the Implementation Self‐efficacy for EBP (ISE4EBP) scale found a mean difference of 13.06 (2.84, 23.28 95% CI, *p* < 0.0123) between the baseline and post‐intervention (Tucker et al. 2022).

Qualitative findings were reported in four studies (Caramanica and Spiva 2018; Gifford et al. 2014; Lavoie‐Tremblay et al. 2012; Proctor et al. 2019). Improvements in leadership competencies were described across studies, namely improvements in the nurse managers' knowledge (Caramanica and Spiva 2018; Proctor et al. 2019), skills, beliefs, and attitudes (Caramanica and Spiva 2018). Improvements in evidence‐based practice implementation leadership competencies were recognized through the activity of the nurse managers, their improved ability to engage relevant stakeholders (Caramanica and Spiva 2018; Lavoie‐Tremblay et al. 2012; Proctor et al. 2019).

## 4 Discussion

This systematic review and meta‐analysis synthesized evidence from six studies on the effectiveness of educational interventions improving nurse leaders' competence in EBP implementation leadership, suggesting that educational interventions can significantly enhance nurse leaders' competence. They have a positive influence on the staff and the organization through improved organizational readiness and implementation climate. Interventions included in this systematic review were diverse in their key components and delivery. Yet, each of the interventions was characterized as complex, having multiple components.

The meta‐analysis demonstrated substantial improvements in nurse leaders' EBP competence (SMD = 1.76) and knowledge (SMD = 1.05) following educational interventions. Suggesting that structured, theory‐informed programs can be effective in building foundational skills necessary for EBP implementation leadership. This has been demonstrated in undergraduate education (Portela Dos Santos et al. 2022). Improved knowledge provides the cognitive basis for applying EBP strategies in complex organizational context. Yet, studies constantly report challenges in EBP knowledge among nurse leaders (Mathew et al. 2024). Furthermore, the application of that knowledge in form of competence reflects not only increased familiarity with EBP principles but also the ability to integrate EBP into decision‐making and foster a supportive culture for implementation. This has been highlighted in qualitative work regarding the essential competencies for nurse managers in EBP implementation leadership (Chen et al. 2024).

Implementation of evidence‐based practice, often regarded as a major hurdle for evidence‐based practice, can be potentially tackled through leadership (Farahnak et al. 2020). Yet, implementation leadership has not been explicitly considered as a key competence in the American Organization for Nursing Leadership (AONL) core competencies for nurse leadership (Hughes et al. 2022). This meta‐analysis demonstrated that implementation leadership, as well as organizational readiness and implementation climate, can be enhanced with educational interventions. This finding is in accordance with previous research that has shown implementation leadership behaviors create a favorable implementation climate in units and organizations, improving the implementation of EBP (Williams et al. 2020). However, the sustainability of the educational intervention effects on implementation climate and other outcomes remains unknown, as only one study (Tucker et al. 2022) reported follow‐up measures. Therefore, further studies should assess the sustainability of the effects.

The outcomes of organizational and implementation climate showed significant improvements, which suggests that educational interventions for nurse leaders may have an impact on the broader systems. Similarly, the broader system has an impact on how nurse leaders can lead EBP. Organizational level focus, and support for nursing leadership and EBP is notably recognized in the American Nurses Credentialing Center (ANCC) Magnet Recognition Program, The Advancing Research and Clinical practice through close Collaboration (ARCC) Modell, Iowa Model of Evidence‐Based Practice, as well as other international and national programs. Nevertheless, these broader organizational elements do not necessarily require accreditation but can be integrated into the leadership educational interventions themselves. Such examples are studies conducted outside of the nursing leadership, including the Leadership and organizational change for implementation (LOCI) conducted in mental health and substance abuse units (Aarons et al. 2024). Given the central role of nurse leaders in shaping clinical environments, the observed improvements in implementation climate suggest that educational interventions may serve as a lever for broader organizational change.

All studies aimed to improve nurse leaders' EBP leadership. However, the certainty of evidence for all outcomes was rated low. Despite this, the interventions could potentially have real world impact in improving the implementation of EBP. None of the studies reported patient‐level outcomes, which highlights a critical gap in the impact of EBP educational interventions, as their goal is to improve the delivery of care and patient outcomes. It is well established that leadership can have a positive effect on patient outcomes (Hult et al. 2023), and that nurse leadership fosters a climate for EBP implementation (Gillaspie et al. 2025). However, no studies have yet shown an association between nurse leaders' EBP implementation leadership and improved patient outcomes. Investigating this is essential because leadership in EBP is presumed to influence clinical decision‐making and adherence to best practices (Sevy Majers and Warshawsky 2020). Without empirical evidence linking EBP leadership to measurable improvements in care quality, such as safety indicators or patient satisfaction, the justification for investing in EBP leadership development remains incomplete. Establishing this would validate the strategic importance of EBP leadership, guide resource allocation and policy decisions aimed at improving healthcare delivery.

### 4.1 Limitations

The overall certainty of evidence was low, warranting cautious interpretation. Most studies included in the review were non‐randomized, making them particularly vulnerable to confounding factors and selection bias. The included studies had small sample sizes with large confidence intervals, reducing the reliability of the effect estimates. The educational interventions were heterogeneous with a large variability in the intervention components, outcome measures, and follow‐up durations limiting their comparability. Sensitivity and subgroup analyses were not conducted because the number of studies per outcome was very small (2–3), and removing or splitting studies would yield unstable estimates. The meta‐analysis may be subject to significant limitations and bias due to heterogeneity. However, we believe it still offers insight into the direction of effect and provides additional information for readers.

Publication bias cannot be ruled out. Educational interventions targeting nurse leaders may be more likely to be published when positive outcomes are observed, introducing a risk of selective reporting. Additionally, organizational training programs often remain internal and unpublished, which could lead to missing evidence and overestimation of intervention effects. Although we conducted comprehensive searches across multiple databases and trial registries, limitations remain, including possible language restrictions and incomplete coverage of gray literature. Nurse leaders in administrative positions, which was the focus of this systematic review, are not the only leaders in facilitating EBP across organizations; other leaders, including informal leaders, researchers, and educators, have an important role in EBP implementation (Castiglione et al. 2025). This may have contributed to an incomplete evidence base. Therefore, the findings need to be interpreted with caution.

### 4.2 Linking Evidence to Action

Educational interventions for nurse leaders show promise in strengthening EBP implementation leadership competencies and improving organizational readiness and implementation climate. These findings highlight the strategic importance of incorporating structured, theory‐informed educational programs into leadership development. Such integration can enhance individual leadership capacity, foster supportive environments for EBP adoption, and ultimately contribute to improved patient care outcomes. However, the limited number of interventions and reliance on pre‐post designs without extended follow‐up periods indicate a need for more rigorous research to determine long‐term effectiveness and clinical applicability.

**Table:** 

Linking evidence to action
Incorporating structured, theory‐informed education into leadership development can strengthen nurse leaders' EBP implementation leadership competencies. Embedding EBP implementation leadership training into nurse leaders' ongoing professional development, can ensure sustained skill reinforcement and practice change. Equipping nurse leaders with EBP implementation leadership competencies can improve organizational readiness and supportive implementation climates, enabling more successful EBP implementation. Conducting more rigorous, longitudinal evaluations are warranted to determine the long‐term impact of EBP implementation leadership education on clinical outcomes.

## Funding

This work was supported by Stiftelsen Eschnerska Frilasarettet sr foundation.

## Conflicts of Interest

The authors declare no conflicts of interest. The employment of J.V. and R.A. is funded by a grant from *Stiftelsen Eschnerska Frilasarettet sr* foundation administered through the University of Turku. The foundation had no role in the design, execution, or interpretation of the research.

## Supporting information


**Supporting Information: S1:** Search strings to bibliographic databases.

## Data Availability

The data that support the findings of this study are openly available in Open Science Framework at https://osf.io/ngpeh, reference number https://doi.org/10.17605/OSF.IO/NGPEH.
