# Supplementary material for: Enhancing Nurse Leaders Evidence‐Based Practice Implementation Leadership Competence. A Systematic Review and Meta‐Analysis of Educational Interventions
Source: Worldviews Evid Based Nurs. 2026 Jul 13;23(4):e70164. doi: 10.1111/wvn.70164 (PMC13360643; doi:10.1111/wvn.70164)
Supplement: Supplementary file 1 — Supporting Information: S1: Search strings to bibliographic databases. [file WVN-23-0-s001.docx]

Supplementary material: Search strings to bibliographic databases.

| Pubmed | Search string |
| --- | --- |
|  | (“Nurs* leader*” [tiab] OR “Nurs* manag*” [tiab] OR “Head nurs*” [tiab] OR “Charge nurs*” [tiab] OR “Nurs* officer” [tiab] OR “First line manager*” [tiab] OR “Ward manag*”[tiab] OR “Unit manag*”[tiab] OR “Nurs* administrator*”[tiab] OR "Nurse Administrators"[Mesh] OR “Nurs* supervisor”[tiab] OR leadership [tiab] OR "Leadership"[Mesh]) AND (“Evidence-based practice”[tiab] OR "Evidence-Based Practice"[Mesh]) AND  ("Education"[Mesh] OR educatio* [tiab] OR Onboarding [tiab] OR Training [tiab] OR “Evidence-based practice education” [tiab] OR Instruct* [tiab] OR Learn* [tiab] OR “Innova* approaches” [tiab] OR "Curriculum"[Mesh] OR Curriculum [tiab] OR Mentoring [tiab] OR "Mentoring"[Mesh] OR “Organizational support”[tiab] OR “Organisational support”[tiab] OR “Professional development”[tiab] OR “Collaborative approach”[tiab] OR teaching [tiab] OR "Teaching"[Mesh]) AND (“Clinical practice improvement” [tiab] OR Knowledge*[tiab] OR "Knowledge"[Mesh] OR “Organizational culture” [tiab] OR “Organisational culture” [tiab] OR “Behaviour change*” [tiab] OR "Behavior"[Mesh] OR Understanding [tiab] OR Confidence [tiab] OR Skills [tiab] OR Competenc*[tiab] OR attitude* [tiab] OR "Attitude"[Mesh]) |
| CINAHL | MH "Nursing Leaders+" OR TI “Nurse leader*” OR AB “Nurse leader*” OR MH "Nurse Managers+" OR MH "Nursing Management+" OR TI “Nurs* manag*” OR AB “Nurs* manag*” OR MH "Head Nurses" OR TI “Head nurs*” OR AB “Head nurs*” OR MH "Charge Nurses" OR TI “Charge nurs*” OR AB “Charge nurs*” OR TI “Nurs* officer” OR AB “Nurs* officer” OR TI “First line manager*” OR AB “First line manager*” OR TI “Ward manag*” OR AB “Ward manag*” OR TI “Unit manag*” OR AB “Unit manag*” OR MH "Nurse Administrators" OR TI “Nurse administrator*” OR AB “Nurse administrator*” OR MH "Nursing Administration+" OR TI “Nurse supervisor” OR AB “Nurse supervisor” OR TI leadership OR AB leadership OR MH "Nurse Executives+"  AND (MH "Professional Practice, Evidence-Based+" OR TI “Evidence-based practice” OR AB “Evidence-based practice”) AND (MH "Education+" OR TI educatio* OR AB educatio* OR TI onboarding OR AB onboarding OR TI training OR AB training OR TI “Evidence-based practice education” OR AB “Evidence-based practice education” OR TI instruct* OR AB instruct* OR MH "Learning+" OR TI learn* OR AB learn* OR TI “innova* approaches” OR AB “innova* approaches” OR MH "Curriculum+" OR TI curriculum OR AB curriculum OR TI mentor* OR MH "Mentorship" OR AB mentor* OR TI “organizational support” OR AB “organizational support” OR TI “organisational support” OR AB “organisational support” OR TI “professional development” OR AB “professional development” OR TI “collaborative approach” OR AB “collaborative approach” OR MH "Teaching+" OR TI teaching OR AB teaching) AND (TI “clinical practice improvement” OR AB “clinical practice improvement” OR MH "Knowledge+" OR TI knowledge* OR AB knowledge* OR TI “organizational culture” OR AB “organizational culture” OR TI “organisational culture” OR AB “organisational culture” OR MH "Behavioral Changes" OR TI “behaviour change*” OR AB “behaviour change*” OR TI understanding OR AB understanding OR TI confidence OR AB confidence OR TI skills OR AB skills OR MH "Professional Competence+" OR MH "Clinical Competence+" OR TI competenc* OR AB competenc* OR MH "Attitude+" OR TI attitude* OR AB attitude*) |
| Scopus | (“Nurse leader*” OR “Nurs* manag*” OR “Head nurs*” OR “Charge nurs*” OR “Nurs* officer” OR “First line manager*” OR “Ward manag*” OR “Unit manag*” OR “Nurse administrator*” OR “Nurse supervisor” OR leadership) AND (“Evidence-based practice”) AND (educatio* OR Onboarding OR Training OR “Evidence-based practice education” OR Instruct* OR Learn* OR “Innova* approaches” OR Curriculum OR Mentoring OR “Organizational support” OR “Organisational support” OR “Professional development” OR “Collaborative approach” OR teaching) AND (“Clinical practice improvement” OR Knowledge* OR “Organizational culture” OR “Organisational culture” OR “Behaviour change*” OR Understanding OR Confidence OR Skills OR Competenc* OR attitude*) |
| Web of Science | (“Nurse leader*” OR “Nurs manag*” OR “Head nurs*” OR “Charge nurs*” OR “Nurse officer*” OR “nursing officer” OR “First line manager*” OR “Ward manag*” OR “Unit manag*” OR “Nurse administrator*” OR “Nurse supervisor*” OR “leadership”) AND (“Evidence-based practice”) AND (educatio* OR Onboarding OR Training OR “Evidence-based practice education” OR Instruct* OR Learn* OR “Innovative approach*” OR Curriculum OR Mentoring OR “Organizational support” OR “Organisational support” OR “Professional development” OR “Collaborative approach” OR teaching) AND (“Clinical practice improvement” OR Knowledge* OR “Organizational culture” OR “Organisational culture” OR “Behaviour change*” OR Understanding OR Confidence OR Skills OR Competenc* OR attitude*) |
| Cochrane library | (nurse NEXT leader* OR nurse NEXT manag* OR Head NEXT nurs* OR Charge NEXT nurs* OR Nurse NEXT officer OR First NEXT line NEXT manager* OR Ward NEXT manag* OR Unit NEXT manag* OR Nurse NEXT administrator* OR Nurse NEXT supervisor OR leadership) AND (Evidence-based NEXT practice) AND (education* OR Onboarding OR Training OR Evidence-based NEXT practice NEXT education OR Instruct* OR Learn* OR Innovative NEXT approaches OR Curriculum OR Mentoring OR Organizational NEXT support OR Organisational NEXT support OR Professional NEXT development OR Collaborative NEXT approach OR teaching) AND (Clinical NEXT practice NEXT improvement OR Knowledge* OR Organizational NEXT culture OR Behaviour NEXT change OR Understanding OR Confidence OR Skills OR Competenc* OR attitude*) |
